# Shh promotes sweat gland cell maturation in three-dimensional culture

**DOI:** 10.1007/s10561-016-9548-7

**Published:** 2016-02-23

**Authors:** Zhijian Huang, Yunfang Zhen, Wei Yin, Zhourui Ma, Liya Zhang

**Affiliations:** Department of Burn and Plastic Surgery, Children’s Hospital of Soochow University, Suzhou, Jiangsu Province People’s Republic of China; The Center of Diagnosis and Treatment for Children’s Bone Diseases, Children’s Hospital of Soochow University, Suzhou, Jiangsu Province People’s Republic of China; Department of Neurology, Children’s Hospital of Soochow University, Suzhou, Jiangsu Province People’s Republic of China

**Keywords:** Fibroblasts, Shh, Sweat gland tubule-like structure, 3D culture

## Abstract

Sweat gland (SG) cells forming SG tubule-like structures in 3D culture, this is one of the most important methods to identify the biological function of SG cells and stem cells-derived SG-like cells, but also the important way on research of SG regeneration in vitro. In this study, we seeded human fibroblasts and SG cells in gels and used immunohistochemistry to confirm whether SG tubule-like structures formed. Fibroblasts are necessary factor in the process of SG cells maturation and forming SG’s secretory region in 3D culture. Further experimentation revealed that Sonic hedgehog (Shh) was secreted by fibroblasts within the 3D culture. By adding Shh protein to 3D culture, there had more SG tubule-like structures formed. These results suggest that Shh is an important factor during the process of forming SG tubule-like structures in 3D cultures, and adding Shh recommbinant protein could promote SG cell maturation and enhance the efficiency of structure formation.

**Electronic supplementary material:**

The online version of this article (doi:10.1007/s10561-016-9548-7) contains supplementary material, which is available to authorized users.

## Introduction

Sweat glands are one of the most important skin appendages; their main function is to perspire and control body temperature. Sweat gland development is complicated, and the production of sweat glands ceases after birth; once sweat glands are destroyed, they cannot regenerate (Saga 2002). There are millions of burn injury patients every year across the world. Of these patients, approximately 10 % suffer severe full-thickness burns to their skin (Fu et al. 2006). A common treatment for burn injuries is the use of skin allografts to cover the wounds (Cuono et al. 1986; Naoum et al. 2004; Burd and Chiu 2005). These, however, not involved sweat gland, increasing the patient’s pain.

The development of tissue engineering has opened up a new path for the repair of large skin lesions (Fu et al. 2005). The reconstruction of skin that possesses not only the epidermal and dermal portions but also skin appendages is important. Therefore, regenerating the structure of sweat glands is an important clinical problem. Accumulating studies have focused on the regeneration of sweat glands. Researchers have found that bone marrow mesenchymal stem cells (MSCs) can differentiate into sweat gland-like cells in vitro. Following the transplantation of these cells into nude mice or deep burn injury patients, damaged sweat glands were reconstructed (Sheng et al. 2009).

With the recent development of extracellular matrices, the 3D reconstruction of sweat glands in vitro has become possible (Kleinman and Martin 2005). Whether sweat gland cells can form tubule-like structures in 3D culture, this is an important indicator for identification of sweat gland and stem cells-derived sweat gland cells and their biological function; this is also an important method for sweat gland tissue engineering research. A 3D culture system has been established using Matrigel, and sweat gland cells cultured in this system can form tubule-like structures (Li et al. 2013). Furthermore, a collagen type I solution and Matrigel were mixed and epidermal growth factor (EGF)-loaded microspheres were added as both a slow-release depot of growth factors and a delivery vehicle for the SG cells. SG cells in this system could also form sweat gland tubule-like structures (Huang et al. 2010).

However, what factors does fibroblast secrete of this process remain unclear. In this study, we found that fibroblast was an essential factor for sweat gland cell forming tubule-like structures in 3D culture system; we demonstrated that Shh was an important factor for sweat gland cells form tubule-like structures in 3D culture, and was secreted by fibroblasts; we also found that adding extra Shh can enhance the efficiency of structure formation.

## Materials and methods

### Isolation and culture of human sweat gland cells

Infant polydactyly skin samples were obtained from the Children’s Hospital affiliated to Soochow University and were donated with the parents’ informed consent. The process was reviewed and approved by the Institutional Review Board and the Ethics Committee of the First Affiliated Hospital of the PLA General Hospital (Huang et al. 2010). Tissues were washed in phosphate buffered saline (PBS) with penicillin/streptomycin (Gibco) for 10 min at room temperature. After removing subcutaneous fat, the skin was minced into 1 mm^3^ pieces and washed in PBS without penicillin or streptomycin. Then, the pieces of skin were treated with dispase (1 mg/ml; Roche) for 18 h. After the dispase treatment, the epidermis and dermis were separated, using nippers to isolate the dermis from the tissue. The dermis was then treated with collagenase type IV (2.5 mg/ml; Sigma, USA) for 1 h at 37 °C. After digestion, the sweat glands were dissociated from the surrounding collagen and fat and individually removed using a transfer pettorunder an ultraviolet-sterilized phase-contrast inverted microscope (×40).

The SG cells were cultured in a sweat gland cell-medium(SGM), consisting of: Glucose Dulbecco’s modified Eagle’s medium (DMEM-F12, Gibco) supplemented with 10 % FBS (Hyclone), 1 % penicillin/streptomycin (Gibco), 2 mM l-glutamine (Gibco), 10 × insulin-transferring sodium selenite solution (ITS, Gibco), 2 nM/ml triiodothyronine (T3, Sigma), 0.4 mg/ml hemisuccinate hydrocortisone (Sigma) and 10 ng/ml human recombinant epidermal growth factor (EGF, R&D).

### Isolation and culture of human dermal fibroblast cells

After isolating the epidermis and dermis, the dermis was cut into small pieces and treated with collagenase type IV for 1 h at 37 °C. After digestion, the mixture was sifted through mesh. The cell suspension was centrifuged and resuspended in high glucose DMEM (Gibco) supplemented with 10 % FBS.

For co-cultured fibroblasts and SG cells, SG cells have stronger adherent ability than fibroblasts. We used 0.25 % Tyrisin/EDTA (Gibco) to digest co-cultured cells for 1 min, fibroblasts were digested in the suspension but SG cells were still adhere on the dish, then we collected fibroblasts cultured in vitro for future experiment.

### RNA isolation and real-time quantitative PCR

Total RNA was isolated from cells using the RNeasy Mini extraction kit (Qiagen, Courtaboeuf, Germany) according to the manufacturer’s protocol. A total of 1 µg RNA was used for reverse transcription. For PCR analysis, cDNA was reverse transcribed using a Reverse Transcriptase M-MLV kit (TaKaRa). For real time-PCR analysis, cDNA was reverse transcribed using a PrimeScript™RTMaster Mix kit (TaKaRa) according to the manual. The primer mixes were loaded in duplicate with SYBR Green PCR Master Mix and 1 µg (final) cDNA in 96-well plates. Normalization and fold changes were calculated using the ∆ΔCt method (Guenou et al. 2009). The primers used are listed in Supplementary Table S1.

### Histological and immunochemical analysis

Cells were fixed in 4 % paraformaldehyde and blocked for 30 min in 3 % BSA/PBS. All primary antibodies were diluted in blocking buffer (0.1 % BSA/PBS) and incubated with samples overnight at 4 °C. The cells were then incubated with fluorescently labeled secondary antibodies for 1 h. The cell nuclei were counter stained with DAPI (Southern Biotech, Birmingham, USA). All images were captured using a fluorescence microscope (Leica DM 2500). For hematoxylin-eosin staining, the gel was fixed in 4 % paraformaldehyde, dehydrated, and embedded in paraffin.

The primary antibodies used for immunohistochemistry were as follows: anti-EDA (Santa Cruz), anti-EDAR (Santa Cruz), anti-K8 (Abcam), and anti-CEA (eBioscience). The secondary antibodies used were anti-mouse-PE (Santa Cruz) and anti-rabbit-PE (Santa Cruz).

### Three-dimensional culture of SG cells

Collagen I from rat tails (Gibco) and Matrigel (BD) were used to fabricate successive acellular collagen matrix layers on a polycarbonate membrane. The collagen I was prepared as previously described (Carlson et al. 2008). After the acellular collagen matrix was solidified by placing it at room temperature for approximately 20 min, a cellular collagen matrix layer mixed with fibroblasts and SG cells was added on top of the acellular collagen matrix. The mixture was then incubated for 1 h at 37 °C. After the mixture was solidified, Epilife medium (Gibco) supplemented with 50 ng/ml EGF was used to cover the surface of the gel for 21 days, changing the medium every 2 days. After 21 days of culture, the gel was collected for subsequent experiments.

To examine whether Shh influenced the formation of sweat gland tubule-like structures, three groups were designed for comparison: normal 3D culture medium, medium supplemented with 40 ng/ml Shh recombinant protein (Peprotech), or medium supplemented with Shh antagonist, 20 μmol/L cyclopamine (Sigma). During 3D culture, Shh recombinant protein or Shh antagonist was added to both the gel and the culture medium.

### Counting standards for sweat gland-like structures formed by SG cells

To count the number of tubule-like structures formed in the gel, we developed a protocol to quantify these structures. We selected three sections randomly with an interval of at least 100 μm (to avoid the same structure being counted repeatedly). The counting methods were as follows: sweat gland-like structures formed by SG cells are defined as a structure composed of 6–15 cells in one region, with a clear lumen that is similar to the transverse section of normal human SG tissue. The number of structures was confirmed under the optical microscope.

### Fibroblasts and SG cells labeled with GFP reporter gene and sorted from gel

A lentivirus containing the GFP gene (LV-EGFP) was obtained from Sidansai Stem Cell Technology Co. at a titer of 8 × 10^6^ IU/ml. We seeded 2 × 10^5^ cells and added 1 × 10^6^ IU/ml of LV-EGFP with 10 mg/ml Polybrene. The medium with LV was removed after 24 h. When the cells were nearly 80 % confluent, they were observed under a fluorescence microscope to confirm the ratio of GFP positive cells.

After cells were cultured in gel for 3 weeks, the gel was digested by collagenase type IV (2.5 mg/ml) for 1 h at 37 °C. Then suspension with cells and small pieces gel was stewing for 5 min. Cells in supernatant were collected for sorting. GFP-positive cells were sorted by fluorescence-activated cell sorting (FACS) Calibur (Becton–Dickinson, Franklin Lakes, NJ) for the subsequent experiments.

### ELISA analysis and western blot on fibroblasts

Fibroblasts and SG cells co-cultured supernatant was collected for western blot and ELISA analysis. For western blot, 5 ml co-cultured supernatant was collected and enriched by centrifuging with 12,000 rpm for 10 min. Anti-Sonic Hedgehog antibody (Abcam) was used to detect Shh in supernatant. For ELISA analysis, co-cultured supernatant were collected at 1, 2, 3, 4 and 5 day respectively. A Sonic Hedgehog Human ELISA kit (Abcam) was used to detect Shh concentration in supernatant, according to the manual.

## Results

### Sweat gland-like structures formed in 3D culture are influenced by human fibroblasts

**Fig. 1:**
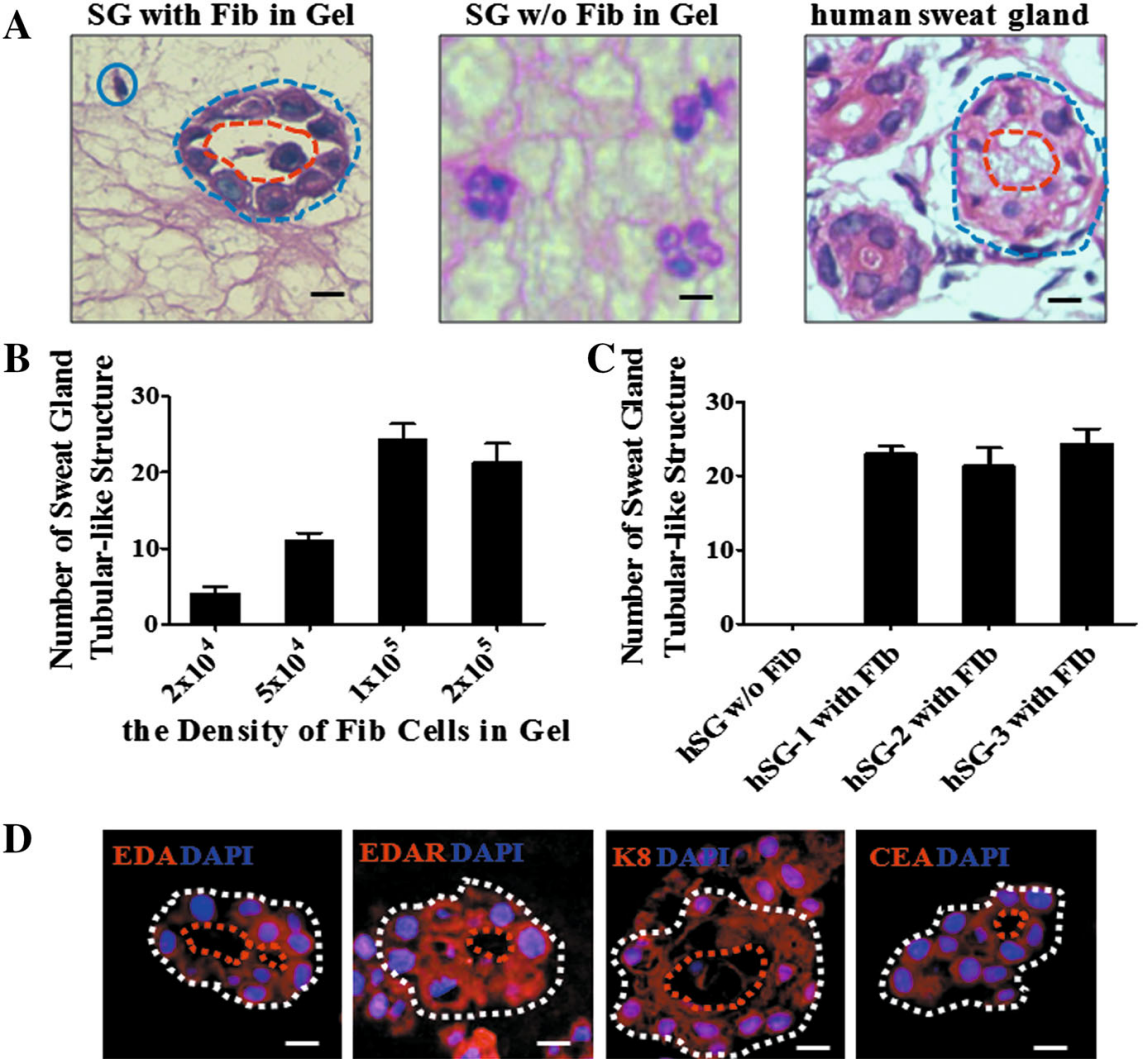
Sweat gland-like structures formed in 3D culture are influenced by human fibroblast cells. **a** H&E images of sweat gland tubule-like structures in gels with or without fibroblasts, and the normal human sweat gland structure. Fibroblasts are indicated with a *blue circle*, the shape of the tubule-like structure with a *blue dashed circle*, and the lumen of the tubule-like structure with a *red dashed circle*. *Scale bar* 15 µm. **b** The number of sweat gland tubule-like structures formed in culture with different densities of fibroblasts with a fixed density of SG cells (see methods for quantification) (n = 3). **c** Comparison of the number of sweat gland tubule-like structures formed by three different fibroblast *cell lines* with a fixed density of SG cells (n = 3). **d** Immunofluorescence staining of sweat gland tubule-like structures formed by fibroblasts and sweat gland cells for sweat gland-related makers: EDA, EDAR, K8 and CEA (*red*); nuclei (*blue*) were stained with DAPI. The shape of the tubule-like structures is indicated by a *white dashed circle* and the lumen of the tubule-like structures by a *red dashed circle*. *Scale bar* 15 µm. (Color figure online)

To find the optimal density of fibroblasts needed for our 3D culture model, we seeded different densities of fibroblasts with 2.5 × 10^4^ SG cells and counted the number of sweat gland tubule-like structures that formed in the gel. As the results show, seeding 1 × 10^5^ fibroblasts formed the most structures (Fig. 1b). This suggests that 1 × 10^5^ fibroblasts is the optimal density for our 3D culture model.

With the optimal density of fibroblasts, we compared three different SG cell lines, counting the number of structures formed in the 3D culture model. As the results show, tubule-like structures did not form without the presence of fibroblasts, whereas with the presence of fibroblasts, the numbers of sweat gland tubule-like structures formed were 23, 22 and 24 (Fig. 1c). The above results showed that the ability to form structures by different sweat gland cell lines was similar, however fibroblasts were necessary during the process of forming sweat gland tubule-like structures in 3D culture.

To observe whether human fibroblasts have a role during 3D culture, human fibroblasts and SG cells were seeded within the gel (in which Matrigel and collagen I were mixed in a 1:1 ratio) for 3 weeks. Immunohistochemical analysis showed that with human fibroblasts in the gel, SG cells could form tubule-like structures organized in a manner similar to skin-derived human sweat gland. However, without fibroblasts in the gel, SG cells could not form tubule-like structures (Fig. 1a).

We also used immunofluorescence staining to confirm the structures formed in the gel (Fig. 1d). SG cells express the sweat gland secretory region makers EDA, EDAR, K8 and CEA. Altogether, these results show that with the help of fibroblasts, SG cells have the ability to form mature tubule-like structures.

### Shh is secreted by human dermal fibroblasts

**Fig. 2:**
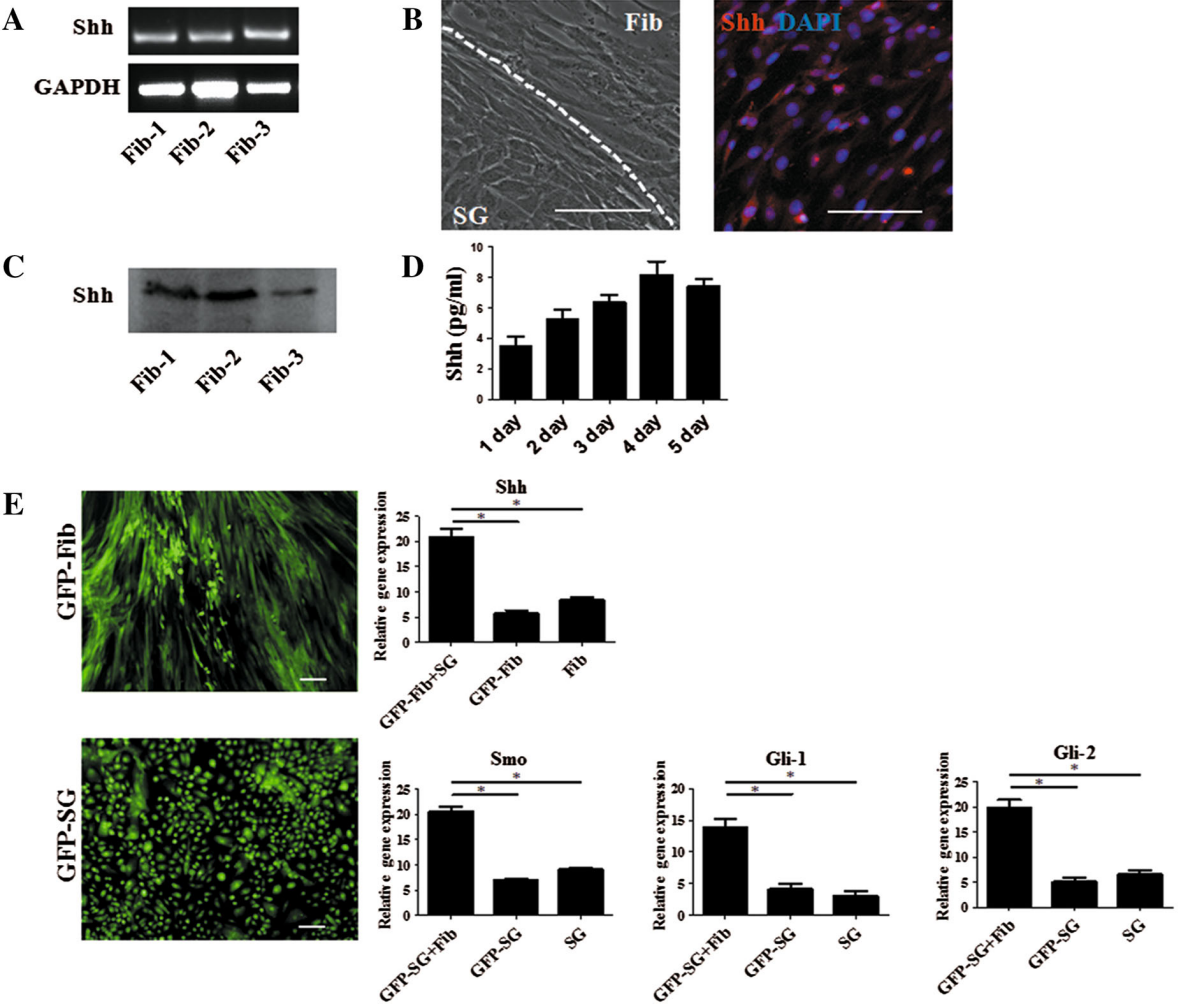
Shh is secreted by human dermal fibroblasts. **a** PCR analysis of three different fibroblast *cell lines* for Shh gene expression. All *three lines* expressed Shh. **b** Morphology of co-cultured fibroblasts and SG cells. Fibroblasts and SG cells were divided by *white dashed circle*. Immunofluorescence staining of fibroblasts for Shh (*red*); nuclei (*blue*) were stained with DAPI. *Scale bar* 40 µm. **c** Western blot analysis of supernatant form fibroblasts co-cultured with SG cells, the result revealed that all *three lines* expressed Shh. **d** ELISA analysis on the collected supernatant also detected the presence of Shh (n = 3). **e** A GFP reporter gene was introduced into fibroblasts and sweat gland cells using lentivirus. Cells were observed under a fluorescence microscope, *Scale bar* 40 µm. For the fibroblasts, quantitative analysis of Shh mRNA expression (GFP-Fib cells co-cultured with SG cells in the gel, without SG cells in the gel and fibroblast cells). For the SG cells, quantitative analysis of mRNA expression of Smo, Gli-1 and Gli-2 on SG cells (GFP-SG cells co-cultured with fibroblasts, without fibroblasts and SG cells). (Color figure online)

During the development of sweat glands, the Shh pathway is important for the formation of the secretory region of the sweat gland (Cui et al. 2014). To find the source of Shh in our 3D culture model, we first performed RT-PCR on primary fibroblasts from dermal. We found that three different lines of fibroblasts all expressed Shh (Fig. 2a). Then we separated fibroblasts which co-cultured with SG cells and cultured those fibroblasts. Immunofluorescence analysis confirmed the presence of Shh protein in fibroblasts (Fig. 2b). To confirm the presence of Shh protein, we collected supernatant form fibroblasts co-cultured with SG cells in vitro for western blot and ELISA analysis. The result demonstrated that Shh was detected in the supernatant (Fig. 2c, d).

To further confirm that fibroblasts secrete Shh in the gel culture, a GFP reporter gene was introduced into fibroblasts using a lentiviral vector to trace the cells in subsequent experiments. Observing the GFP lentivirus-treated fibroblasts (GFP-Fib) under a fluorescence microscope indicated that almost all the cells were labeled with GFP (Fig. 2e). We then used these GFP-Fib cells to do the 3D culture for SG cells. After 3 weeks, GFP-Fib cells were digested from the gel and sorted based on GFP fluorescence. Real time PCR analysis was carried to detect Shh gene expression in fibroblasts, compared with GFP-Fib cells cultured in the gel without SG cells and fibroblasts not cultured in the gel. The results showed that GFP-Fib cells in the gel with SG cells had a higher expression of Shh. Next, we labeled SG cells with GFP-lentivirus (GFP-SG) and observed these cells under a fluorescence microscope to ensure efficient GFP labeling. GFP-SG cells were then seeded in a gel with fibroblasts. After 3 weeks in 3D culture, the GFP-SG cells were digested and sorted for real-time PCR analysis, compared with GFP-SG cells cultured in the gel without fibroblasts and SG cells not cultured in the gel. In the Shh pathway, Smoothened (Smo) is the receptor of Shh, Gli-1 and Gli-2 is downstream Shh pathway genes (Ingham and Placzek 2006). The real time PCR analysis results showed that GFP-SG cells had a higher expression of Smo, Gli-1 and Gli-2.

The above data suggest that Shh is secreted by fibroblasts within the 3D culture. Fibroblasts are likely to interact with SG cells within the gel; fibroblasts secrete Shh, which binds to its receptor Smo on the SG cells, thus activating the Shh pathway, promote the formation of sweat gland tubule-like structures. The SG cells can then, in turn, stimulate the fibroblasts to secrete more Shh and act on SG cells. Those data also suggest that during 3D culture, co-cultured with SG cells was conducive to fibroblasts secrete Shh; and co-cultured with fibroblasts, Shh receptor Smo was higher expressed on SG cells.

### Shh promotes SG cell maturation and enhances the efficiency of structure formation

**Fig. 3:**
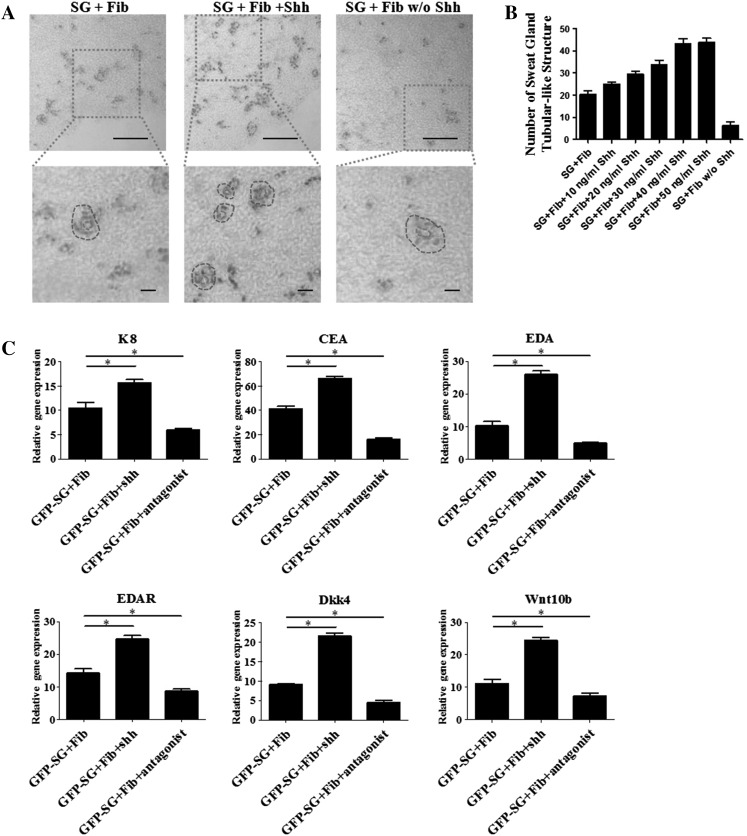
Shh promotes SG cell maturation and enhances the efficiency of structure formation. **a** H&E images of sweat gland tubule-like structures in the gel for three groups (normal 3D culture medium, medium supplemented with Shh protein, or Shh antagonist). The *shape* and the lumen of the tubule-like structure are indicated by a *blue dashed circle* and a *red dashed circle*, respectively. *Scale bar* 15 µm. **b** The number of sweat gland tubule-like structures formed in the three medium conditions (see methods for quantification, n = 3). The Shh protein added in the medium were 10, 20, 30, 40, 50 ng/ml respectively. **c** Quantitative analysis of mRNA expression of sweat gland development-related makers (EDA, EDAR, K8, CEA) and EDA/EDAR pathway downstream gene (Dkk4, Wnt10b) of the three different groups (n = 3). (Color figure online)

To examine whether Shh influences the formation of sweat gland tubule-like structures, we compared three experimental groups (normal 3D culture medium, and medium supplemented with either recombinant Shh protein or a Shh antagonist). The concentration of Shh was optimized via concentration titration. We used different concentration of Shh during 3D culture, and counted the numbers of sweat gland tubule-like structure formed. The results indicated that the number of structure was related with concentration of Shh and 40 ng/ml was the optimal concentration (Fig. 3b).

After 3 weeks of culture, immunohistochemistry analysis was used to confirm the effect of Shh. The results showed that when Shh was added, more tubule-like structures were formed compared with normal 3D culture. Addition of the Shh antagonist, it was hard to find any tubule-like structures (Fig. 3a). The numbers of sweat gland tubule-like structure formed in 3D culture are shown in Fig. 3b. After 3 weeks, To detect gene expression of SG cells in the gel, GFP-SG cells were digested and sorted for real-time PCR analysis. The results showed that, with the addition of Shh, the expression of sweat gland-related genes (K8, CEA, EDA, EDAR) was significantly enhanced. The EDA/EDAR pathway downstream genes Dkk4 and Wnt10b also had a higher expression, whereas with the addition of a Shh antagonist, the expression of these genes was reduced (Fig. 3c). The above data suggest that Shh is an important factor during the formation of sweat gland tubule-like structures in 3D culture; it can promote SG cell maturation and can enhance the efficiency of structure formation by adding extra Shh recombinant protein.

## Discussion

In this study, we used a 3D culture model in vitro to confirm the effect of fibroblasts on the formation of sweat gland tubule-like structures. With the help of fibroblasts, sweat gland cells could form tubule-like structures. We demonstrated that fibroblasts secrete Shh in the 3D culture and that fibroblasts interact with SG cells when co-cultured in the gel. We also found that Shh was an important factor during the process of structure formation, promoting SG cells maturation and adding extra Shh can enhance the efficiency of structure formation.

Fibroblasts have an interesting relationship with cells in the skin. In the study of epidermis reconstitution, the number of fibroblasts within the collagen matrix was a critical factor for the establishment of the epidermis (Ghalbzouri et al. 2002), and fibroblasts assisted in the reconstitution of a stratified epidermis (Biedermann et al. 2010). Fibroblasts were also shown to have an important role in wound healing (Bartold and Raben 1996; Werner et al. 2007; Spiekstra et al. 2007). During the development of sweat gland, in the first place a cellular bud of gland grow into the mesenchyme form the duct of sweat gland, then the duct continue down grow into the mesenchyme and the terminal part coil form the secretory part of sweat gland (Cui et al. 2014). Before the duct and the secretory part form, fibroblasts are already existed in dermal, and provide the environment which is needed by duct and secretory part formation. Our research demonstrated that fibroblast was a necessary factor during the process of forming sweat gland tubule-like structures in 3D culture. This was similar to the previous research that fibroblast played an important role in development of skin.

Our studies also find that fibroblasts secrete Shh in the 3D culture and promote sweat gland cells form tubule-like structures, and fibroblasts have interacted with SG cells During sweat gland development, it has been demonstrated that EDA/EDAR signaling is important during the development of sweat gland (Mustonen et al. 2003; Tucker et al. 2000; Cui et al. 2009). Shh is downstream of EDA/EDAR and a key to the development of several skin appendages (Chuong et al. 2000). In hair follicles, Shh is essential for the normal advancement beyond the hair germ stage of development (Chiang et al. 1999); in salivary glands, Shh is required for the late stage of branching morphogenesis (Jaskoll et al. 2004); and in sweat glands, Shh is required for the final formation of the secretory region (Cui et al. 2014). These previous findings are consistent with our study that Shh can promote sweat gland cells form tubule-like structures in the 3D culture and enhance the efficiency of sweat gland tubule-like structure formation.

Tissue engineering has developed rapidly in recent years, providing many benefits for patients (Griffith and Naughton 2002; Böttcher-Haberzeth et al. 2010; Priya et al. 2008; Groeber et al. 2011). Future developments in skin tissue engineering should include the addition of skin appendages. Based on our research, we can enhance the efficiency of sweat gland formation in 3D culture; we can optimize the identification of functional sweat gland; we also provide a model for drug test on sweat gland in vitro.

## Electronic supplementary material

Below is the link to the electronic supplementary material.

Table S1. PCR and real time PCR primers used in this study. Shh is an important gene during the formation of secretory region of sweat gland, Smo is the receptor of Shh, Gli-1 and Gli-2 is downstream Shh pathway genes, K8 is sweat gland secretion portion gene, CEA is sweat gland cell specific gene, EDA and EDAR are the sweat gland development related genes, Dkk4 and Wnt10b are the downstream genes of EDA/EDAR pathway, and use GAPDH as beta-actin. (TIFF 2112 kb)
