# Supplementary figures and images for: Shh promotes sweat gland cell maturation in three-dimensional culture
Source: Cell Tissue Bank. 2016 Feb 23;17:317–25. doi: 10.1007/s10561-016-9548-7 (PMC4882370; doi:10.1007/s10561-016-9548-7)

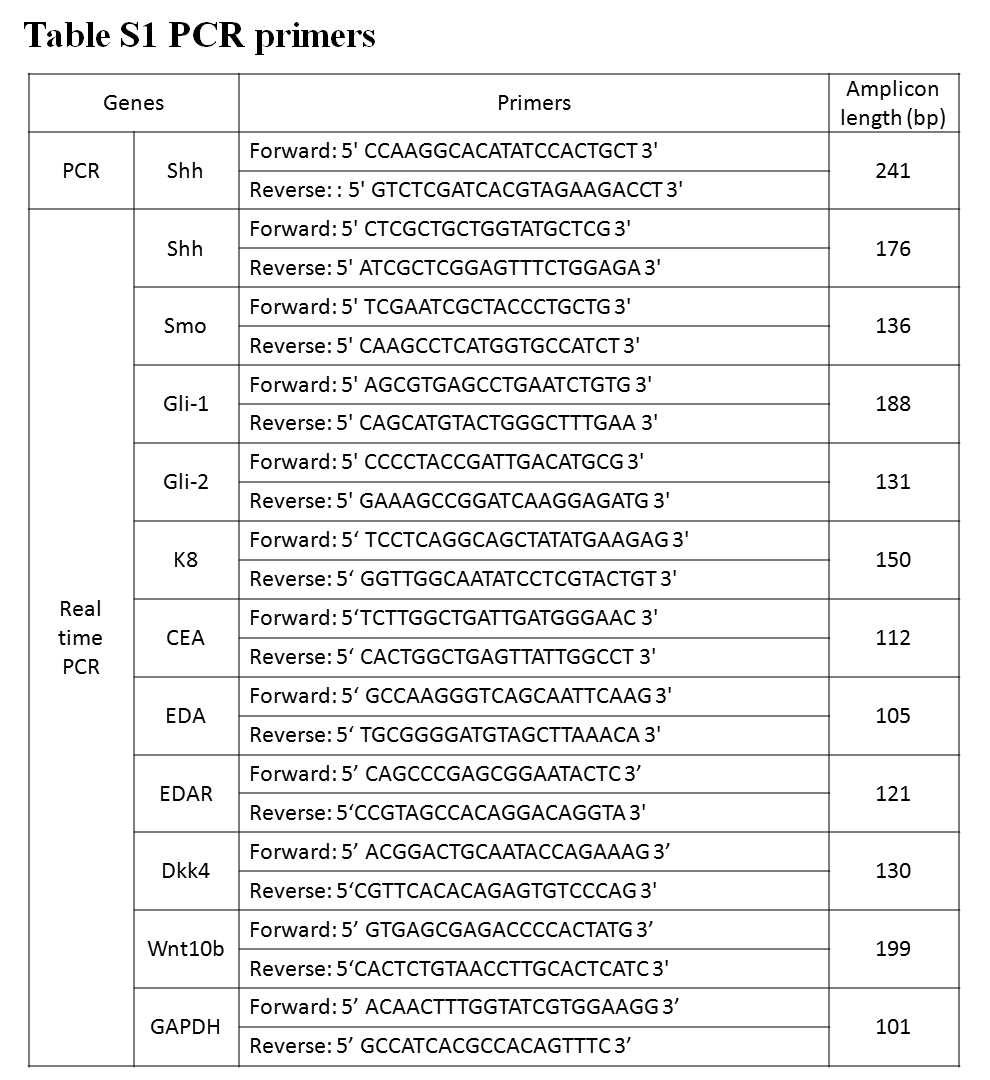

Supplement: Supplementary file 1 — Table S1. PCR and real time PCR primers used in this study. Shh is an important gene during the formation of secretory region of sweat gland, Smo is the receptor of Shh, Gli-1 and Gli-2 is downstream Shh pathway genes, K8 is sweat gland secretion portion gene, CEA is sweat gland cell specific gene, EDA and EDAR are the sweat gland development related genes, Dkk4 and Wnt10b are the downstream genes of EDA/EDAR pathway, and use GAPDH as beta-actin. (TIFF 2112 kb) [file 10561_2016_9548_MOESM1_ESM.tif]
